# Synesthesia: an introduction

**DOI:** 10.3389/fpsyg.2014.01414

**Published:** 2014-12-15

**Authors:** Michael J. Banissy, Clare Jonas, Roi Cohen Kadosh

**Affiliations:** ^1^Department of Psychology, Goldsmiths, University of LondonLondon, UK; ^2^School of Psychology, University of East LondonLondon, UK; ^3^Department of Experimental Psychology, University of OxfordOxford, UK

**Keywords:** synaesthesia/synesthesia, multisensory, sensory substitution, perception, mirror-touch, grapheme-color synaesthesia, lexical-gustatory synaesthesia, cross-modal correspondence

Synesthesia is a rare experience where one property of a stimulus evokes a second experience not associated with the first. For example, in lexical-gustatory synesthesia words evoke the experience of tastes (Ward and Simner, 2003). There are at least 60 known variants of synesthesia (Day, 2013), including reports of synesthetic experiences of color (Baron-Cohen et al., 1987), taste (Ward and Simner, 2003), touch (Ward et al., 2008), and sound (Saenz and Koch, 2008). The lower bound prevalence of the condition is considered to be approximately 4% (Simner et al., 2006). While synesthetic experiences have been documented since the 1800s (Jewanski et al., 2009), it is only in the last few decades that the authenticity of synesthetic experiences and mechanisms that contribute to them has been explored in depth (Ward, 2013). This resurgence in research has led to developments in our understanding of mechanisms that contribute to the synesthetic experience and the use of synesthesia as a unique experimental preparation to inform us about typical models of cognition and perception (e.g., Cohen Kadosh and Henik, 2007; Simner, 2007; Bargary and Mitchell, 2008; Rouw et al., 2011). This has also resulted in many open questions and debates, several of which are touched upon in this research topic. Specifically, this research topic is focused around the following themes: What constitutes synesthesia and how does it relate to typical cross-modal interactions? What mechanisms contribute to synesthetic experiences? Are there broader cognitive and perceptual traits associated with synesthesia, and what mechanisms mediate their relationship? In total, there are 20 articles, each addressing at least one of these themes.

## What constitutes synesthesia and how does it relate to typical cross-modal interactions?

Several authors focus on discussing what synesthesia is and how it relates to typical cross-modal interactions. Mylopoulos and Ro (2013) provide a critical review of methods used for understanding and classifying synesthesia and provide a set of markers to aid in distinguishing synesthesia from other psychological phenomena. Marks and Mulvenna (2013) provide an interesting opinion article on cases that border on traditional forms of synesthesia and discuss whether these forms do or do not constitute forms of synesthesia. Similarly, Deroy and Spence (2013) discuss the notion of “induced synesthesia,” arguing that current attempts to induce synesthesia may not be evidentially linked to developmental synesthesia.

Moos et al. (2013) touch on the theme of how synesthesia relates to typical cross-modal interactions, reporting findings on color and texture associations in voice induced synesthesia, suggesting common underlying mechanisms in cross-modal associations between synesthetes and non-synesthetes. Additionally, Rouw et al. (2014) examine the relationship between synesthetic and non-synesthetic cross-modal representations by assessing color associations for days and letters across different languages.

Haigh et al. (2013) examine the acuity of visual-to-auditory sensory substation and discuss whether visual imagery evoked by the device is a form of or synthetic synesthesia. Similarly, Simner (2013) contributes to themes of what mechanisms contribute to synesthetic experiences and what constitutes synesthesia by discussing the role of visual mental imagery in different types of synesthetes. In her thoughtful discussion, she suggests that differences between projector (for whom synesthetic experiences are projected onto external objects) and associator (for whom synesthetic experiences appear in the “minds eye”) synesthetes may emerge from individual differences in visual mental imagery.

## What mechanisms contribute to synesthetic experiences?

In addition to Simner's (2013) proposal that synesthetic experience is associated with individual differences in visual mental imagery, several other articles also address the mechanisms underlying synesthesia. Gertner et al. (2013) propose that numerical synesthesia is more than a symbol-induced phenomenon, and may also be induced by non-symbolic magnitudes. Perry and Henik (2013) report an experiment addressing emotional conflict sensations evoked in synesthesia when synesthetic photisms and veridical experiences conflict (e.g., when a numeral is presented in the wrong color). They discuss their findings in relation to emotional experience in synesthesia and the extent to which synesthesia may be used as a vehicle to inform us about emotional processing in the wider population. Dael et al. (2013) also address affect in synesthesia by providing a thoughtful review article on affect-related synesthesias and underlying mechanisms. Additionally, Jarick et al. (2013) examine vantage point preference and visual dominance in a time-space synesthesia, reporting that their synesthete is able to reverse her perspective on “time.”

Moving to neural mechanisms, O'Hanlon et al. (2013) provide a research article examining structural and functional brain correlates of grapheme-color synesthesia. Colizoli et al. (2013) report a single case brain imaging study of lexical-gustatory and sound-gustatory synesthesia. Additionally, Luke and Terhune (2013) provide an important review of the induction of synesthesia with chemical agents, highlighting the potential role of the serotonergic system in synesthesia.

Finally Chiou and Rich (2014) discuss the role of conceptual knowledge in understanding synesthesia. Using a “hub and spokes” approach they present a model of synesthesia in which the inducer and concurrent are linked within a conceptual-level representation.

## Are there broader cognitive and perceptual traits associated with synesthesia, and what mechanisms mediate their relationship?

Several authors highlight that synesthetes show different performance on tasks that are not directly related to their synesthetic experience. For example, Chun and Hupé (2013) address the relationship between synesthesia and other perceptual experiences by examining the prevalence of mirror-touch (Banissy et al., 2009) and ticker tape (Day, 2005) experiences in synesthesia. Bouvet et al. (2014) report an interesting case study involving an autistic individual who possesses savant abilities in addition to absolute pitch and synesthetic-like associations. They discuss the case in relation to the role of veridical mapping in autism, absolute pitch and synesthesia. Nielsen et al. (2013) examine and discuss the influence of synesthetic perceptions on sexual experience.

In relation to cognitive differences between synesthetes and non-synesthetes, Ward et al. (2013) address the relationship between grapheme-color synesthesia and enhanced recognition memory by comparing visual recognition memory in grapheme-color and lexical gustatory synesthesia. They show that grapheme-color synesthetes show enhanced visual recognition memory, but this is not found for lexical-gustatory synesthesia. Meier and Rothen (2013) investigate whether synesthesia is associated with a particular cognitive style, and provide evidence to suggest that grapheme-color synesthetes show a preference for a verbal and a specific visual cognitive style.

## Concluding remarks

In summary, this Research Topic provides a novel collection of articles on synesthesia addressing a range of issues. We envisage that many of these will provide productive new research areas, and conceptual frameworks for the future study of synesthesia and related processes.

### Conflict of interest statement

The authors declare that the research was conducted in the absence of any commercial or financial relationships that could be construed as a potential conflict of interest.
